# Supplementary material for: The microbiome biomarkers of pregnant women’s vaginal area predict preterm prelabor rupture in Western China
Source: Front Cell Infect Microbiol. 2024 Oct 31;14:1471027. doi: 10.3389/fcimb.2024.1471027 (PMC11560878; doi:10.3389/fcimb.2024.1471027)
Supplement: Supplementary file 1 [file DataSheet1.zip › compare_1/Community/Pieplot/order/group/P.order.pieplot.pdf]

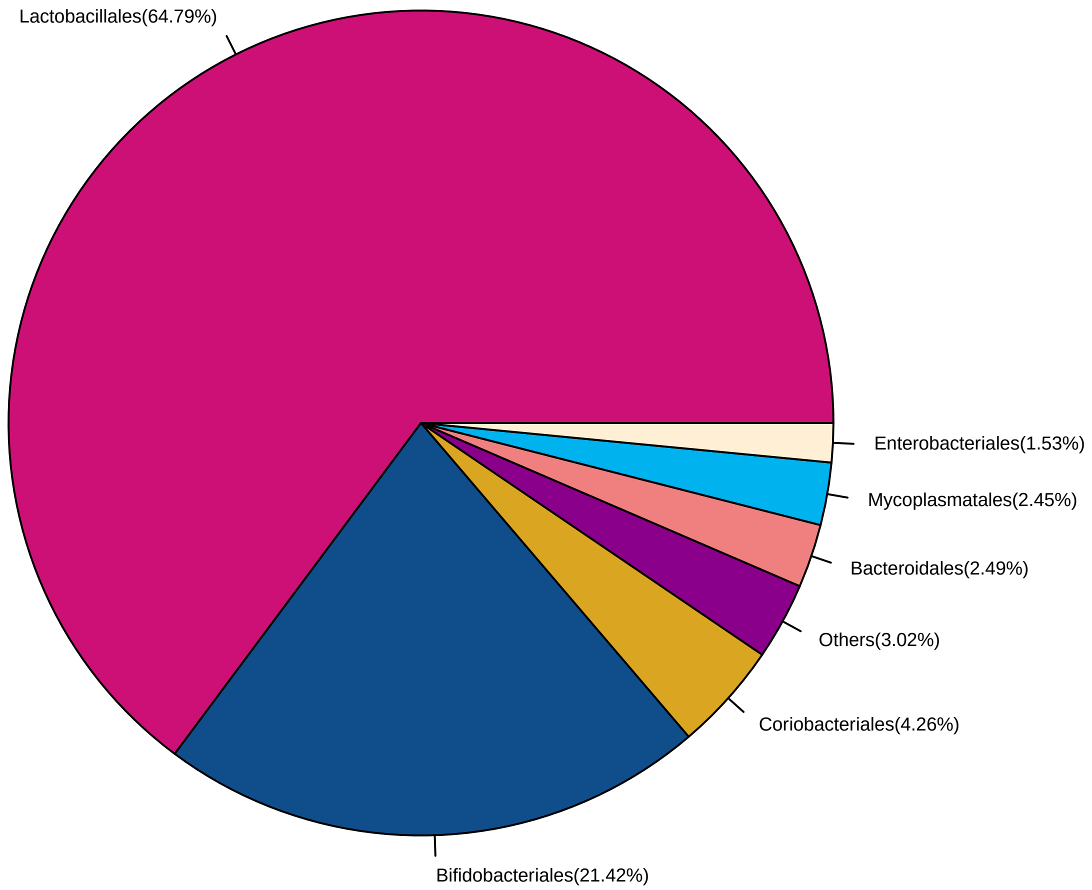
Lactobacillales(64.79%)
Enterobacteriales(1.53%)
Mycoplasmatales(2.45%)
Bacteroidales(2.49%)
Others(3.02%)
Coriobacteriales(4.26%)
Bifidobacteriales(21.42%)
